# Development and Validation of a Risk Model for Predicting Adverse Drug Reactions in Older People during Hospital Stay: Brighton Adverse Drug Reactions Risk (BADRI) Model

**DOI:** 10.1371/journal.pone.0111254

**Published:** 2014-10-30

**Authors:** Balamurugan Tangiisuran, Greg Scutt, Jennifer Stevenson, Juliet Wright, G. Onder, M. Petrovic, T. J. van der Cammen, Chakravarthi Rajkumar, Graham Davies

**Affiliations:** 1 Discipline of Clinical Pharmacy, School of Pharmaceutical Sciences, Universiti Sains, Malaysia, Pulau Pinang, Malaysia; 2 Institute of Pharmaceutical Science, King’s College London, London, United Kingdom; 3 Department of Medicine, Brighton and Sussex Medical School, Brighton, United Kingdom; 4 Department of Geriatrics, Catholic University of the Sacred Heart, Rome, Italy; 5 Department of Geriatrics and Gerontology, Ghent University Hospital, Ghent, Belgium; 6 Department of Internal Medicine, Section of Geriatric Medicine, Erasmus University Medical Center, Rotterdam, The Netherlands; 7 Faculty of Industrial Design Engineering, Delft University of Technology, Delft, The Netherlands; Kyushu University, Japan

## Abstract

**Background:**

Older patients are at an increased risk of developing adverse drug reactions (ADR). Of particular concern are the oldest old, which constitute an increasingly growing population. Having a validated clinical tool to identify those older patients at risk of developing an ADR during hospital stay would enable healthcare staff to put measures in place to reduce the risk of such an event developing. The current study aimed to (1) develop and (2) validate an ADR risk prediction model.

**Methods:**

We used a combination of univariate analysis and multivariate binary logistic regression to identify clinical risk factors for developing an ADR in a population of older people from a UK teaching hospital. The final ADR risk model was then validated in a European population (European dataset).

**Results:**

Six-hundred-ninety patients (median age 85 years) were enrolled in the development stage of the study. Ninety-five reports of ADR were confirmed by independent review in these patients. Five clinical variables were identified through multivariate analysis and included in our final model; each variable was attributed a score of 1. Internal validation produced an AUROC of 0.74, a sensitivity of 80%, and specificity of 55%. During the external validation stage the AUROC was 0.73, with sensitivity and specificity values of 84% and 43% respectively.

**Conclusions:**

We have developed and successfully validated a simple model to use ADR risk score in a population of patients with a median age of 85, i.e. the oldest old. The model is based on 5 clinical variables (≥8 drugs, hyperlipidaemia, raised white cell count, use of anti-diabetic agents, length of stay ≥12 days), some of which have not been previously reported.

## Introduction

Over the next 50 years, many societies throughout the world are set to face an ‘*ageing population*’, with its associated burden of disease and disability. In the US, the proportion of the population aged 65 years or over in 1998 was 13% [1]. By 2012 that proportion had risen to 15%, and is projected to rise to 22% by the year 2060 [2]. The oldest old (those aged 85 or over) will also see a considerable rise in the size of their population, which is set to increase from 6 million in 2012 to 18 million in 2060 (an increase of just under 200%). The picture in the UK is similar. Here, the oldest old (>85 years) will see the largest relative rise in their population of over 5 times between the years 1985 and 2035 [3].

Those aged over 65 are the largest consumers of healthcare, and the recipients of the majority of prescribed medication. It is not surprising therefore that older people suffer more adverse drug reactions (ADR) than younger adults, and are 7 times more likely to require hospitalisation due to an ADR [4]. They are also more likely to suffer an ADR during inpatient stay. This obviously places an extreme pressure on healthcare systems, with medical teams having to treat the consequences of ADRs in addition to the primary pathology.

A considerable amount of work has been conducted in the past 30 years to develop risk prediction models for disease, so that pharmacological, and lifestyle interventions can be targeted to those in most need (for example the Framingham Heart Study) [5]. In 1997, McElnay *et al* identified several risk factors but their final model, predicting adverse drug events (defined as ADRs and ineffective treatment), had low accuracy scores and so further development was halted [6]. In recent years, risk prediction models for ADRs have begun to emerge, offering healthcare practitioners a potential tool to assist clinical and therapeutic decision making, especially in high-risk patient groups such as the over 65s. Trivalle *et al* used data from Parisian rehabilitation hospitals to create a model which focus’ purely on medication as risk variables [7]. In 2010, the GerontoNet ADR risk score was published which used a range of clinical and drug-related risk factors, drawn from an historical database of patients in Italy. This model was then externally validated using data from across four European countries [8]. In their validation study, patients had a mean age of 80.3 years (±7.6), and the Area Under the Curve was 0.71 (0.68–0.73). O’Connor et al recently applied the Gerontonet tool prospectively to a separate cohort of acutely ill patients and found that it correctly predicted ADR risk in 62% of patients. The study also identified additional predictors (e.g. renal function, age, potentially inappropriate medicines, number of medicines) which they suggest should be included in future tools [9].

Although the majority of risk prediction models developed in this area have been validated to some extent, the number of events detected has been relatively small. They have also, in general, included patients with a wide age-range, and the final models are arguably impractical for everyday clinical use. The current study therefore aimed to (1) develop, and (2) validate (using a 4-stage process) an ADR risk prediction model. Our objectives were to produce a model that is robust and generalizable across populations, simple to use, and appropriate for predicting ADR risk in the oldest old (≥85 years). The model was developed from a prospective and comprehensive assessment of ADR events in a teaching hospital in Brighton, UK. It was then externally validated using data collected from centres in Italy, Belgium, the UK, and the Netherlands.

## Methods

### The Brighton Dataset

All patients aged ≥65 years that were admitted to one of 4 wards (2 elderly care and 2 stroke) at Brighton and Sussex University Hospitals NHS Trust (BSUHT) over two 3-month study periods (January to March in 2007 and 2008) September 2008 to February 2009) were systematically enrolled into the study. However, the elderly care wards at BSUHT used in this study only accept patients ≥80 years old, which ensured that the majority of patients enrolled in our study were the oldest old. Most patients were admitted through either the accident and emergency department or the acute medical and surgical assessment unit. Patients were subsequently excluded if they met one of the following exclusion criteria: admitted following self-poisoning, transferred to another ward during the weekend, admitted and discharged during the weekend, died within 24 hours of admission, or medical notes were not available for further investigation. All remaining admissions (690) were followed until discharge, and form the ***B*** *righton* ***AD*** *R* ***RI*** *sk Study Dataset* (BADRI) [10].

### Assessing the presence of an Adverse Drug Reaction (ADR)

For the purpose of this study we adopted the Edwards and Aronson definition of an ADR: an appreciably harmful or unpleasant reaction, resulting from an intervention related to the use of a medicinal product, which predicts hazard from future administation and warrants prevention or specific treatment, or alteration of the dosage regimen, or withdrawal of the product.’ [11]. Suspected ADRs were first identified by the primary investigator (BT) using the trigger tool methodology which was first developed by Classen [12], then later scrutinized and updated by Rozich [13], Resar [14], and Handler [15]. This was conducted through review of procedure notes, emergency department notes, physician progress notes, laboratory reports, physician medication orders on drug charts, nursing flow sheets, hospital discharge records, and multidisciplinary progress notes. Additionally, reports of ADRs from health care-providers and those identified following a review of administrative incident reports concerning ADRs were also included for further review. Patients were however not directly consulted by the primary investigator. All suspected events were then discussed with the attending hospital physician or hospital pharmacist to confirm interpretation. Each event was then reviewed by an independent reviewer (physician) and the primary investigator (pharmacist) to determine the causal relationship between drug intake and the suspected ADR. This was conducted using the Hallas algorithm [16], where each event was classified as either being a definite, probable, possible or unlikely ADR according to the number of predefined criteria that were met. The predefined criteria were: 1) known ADR, 2) reasonable temporal relationship, 3) dechallenge, 4) rechallenge, and 5) no other explanation for the condition. To further strengthen the methodology, confidence about the causality assessment for each event was rated by the reviewers on a 6-point Likert scale devised by Bates and colleagues [17]. The six point Likert scale consists of: 1 little or no confidence; 2, slight-to-moderate confidence; 3, less than 50 percent confidence but a close call; 4, more than 50 percent confident but a close call; 5, strong confidence; and 6, virtually certain. All events classified as definite, probable, or possible, and which received >50% confidence rating (a score of >4) were classified as drug-related and included in the analysis.

### Variables included in the risk prediction model

The following data were collected for each patient included in the *Brighton dataset*: basic demographic data (age, gender, and ethnic origin); admission diagnosis and details of the presenting complaint; details of previous admissions; length of stay; information on social settings (e.g. living alone, smoking and alcohol history); assessment of cognition (Abbreviated Mental Test Score, AMTS); assessment of disability (Barthel Index); biochemical and haematological markers; drug history. We used the WHO International Statistical Classification of Diseases and Related Health Problems to classify comorbidities [18], and renal impairment was taken to be an eGFR <60 ml/min [19].

All data were obtained within 48 hours of admission to the study ward from a combination of a review of the medical notes and through discussion with nursing staff, pharmacists and the medical team attending the patient.

### Statistical analysis

The process of developing the risk prediction model consisted of 2 stages. The first stage involved application of univariate analysis to generate a framework of variables, or predictors, for developing an ADR. Those variables which were found to be statistically significant (p<0.05) were taken forward to the next stage of multivariate analysis. In addition, variables that were identified in other studies as being important predictors of ADR (**Table 1**), but which had p values >0.05 but <0.25 were also included in the multivariate stage (see Hosmer DE, 1989 for further details on this methodology) [20]. Variables which were present in less than 5% of the study population were omitted from the analysis due to the risk of including a statistically significant artefact in the model.

**Table 1:** Predictor variables identified from the univariate analysis stage, and from other studies, only 8 of which were taken forward to the multivariate analysis stage (indicated with *).

Variables identified in univariate analysis
Hyperlipidaemia*
Number of medication ≥8*
Length of stay ≥12 days*
Use of anti-diabetic agents*
High white blood cell count on admission*
Diabetes
Arthritis/osteoarthritis
Antihypertensives
Opioid analgesics
Angiotensin converting enzymes inhibitors
β-blockers
Cardiac glycosides
Anti-infective medicines
**Variables identified in other studies**
History of previous ADR* [8], [28]
Number of co-morbidities ≥4* [29]
Drugs with a narrow therapeutic index* [27], [30]
Congestive cardiac failure [31]
Liver failure [32], [33]

All variables are binary, and not continuous.

Multivariate analysis was undertaken using binary logistic regression analysis. First variables were removed from the model using the backward elimination process. The removal criteria was set at p = 0.10 to allow variables to be retained in the model for as long as possible. The process was then repeated with the forward selection procedure before final confirmation of the model. Selected candidate variables were later assessed for multicollinearity and association to rule out any strong correlation between two or more candidate variables in the final model. A p>0.05 from a χ^2^ test was taken to indicate an association. Following the iteration process, the retained variables were taken forward into the final risk model.

The overall fit of the BADRI risk prediction model to the *Brighton dataset* was assessed using the Hosmer-Lemeshow test, and effect size measured using the Nagelkerke R^2^. The cut-off BADRI score was determined by first calculating sensitivity (proportion of true positives) and specificity (proportion of true negatives) of the model for the range of risk scores (0–5) and then calculating Youden’s Index (J; *Equation 1* *)*. Discrimination of the model was further assessed by an analysis of the area under the operator curve (AUROC), which assesses the ability of the risk score to predict ADR. AUROC indicates how well the model distinguishes patients who do not experience ADR from those with ADR (Bewick et al., 2005). The AUROC value signifies the probability that a patient with an ADR had a higher predicted probability than a patient without ADR. An ideal model would have an AUROC of 1, whereas a random guess would have an AUROC of 0.5 [21].
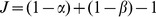
(1)Where (1−α) is Sensitivity and (1−β) is Specificity.

### Validation

The ADR risk prediction model (BADRI risk score) was validated in a separate cohort of in-hospital older adults admitted to four Geriatric or Internal Medicine wards in participating study centres across Europe. These centres, which contribute to the *European Dataset,* were: Catholic University of Sacred Heart, Rome, Italy; Gent University Hospital, Ghent, Belgium; Erasmus University Medical Center, Rotterdam, The Netherlands and BSUH (Brighton, UK). The aim of this part of the study was to evaluate the reliability of BADRI score locally and also in other populations.

Patients admitted to these centres between September 2008 and December 2008 were enrolled and followed up until discharge or death. Exclusion criteria were identical to the Brighton Dataset with the addition of patients receiving current chemotherapy. In accordance with the methods described above, data for all variables included in the model (along with a selection of other variables, e.g. Mini Mental State Examination MMSE) were recorded for each patient, along with details of any suspected ADR. In an attempt to standardise the information being collected from the various European centres, the Naranjo algorithm was used to assess causality of reported ADR in this instance as it has evidence for inter-observer reliability [22]. Only events classified as definite (score, 9–12 points) or probable (score, 5–8 points) were considered to have been drug-related in the validation part of this study. Pair assessment was not conducted for the validation dataset.

As with assessment of the model in the *Brighton dataset*, sensitivity and specificity, Youden’s Index, and AUROC were calculated on the data gathered from the validation cohort.

### Ethics

Ethical approval for conducting the validation component of this research was obtained from Brighton West Research Ethics Committee, UK (reference number: 08/H1111/43). Written informed consent was given by participants (or next of kin/caregiver) for the validation component of this study. For all stages of this research, patient records/information were anonymized and de-identified prior to analysis.

## Results

### Patients

**Figure 1:**
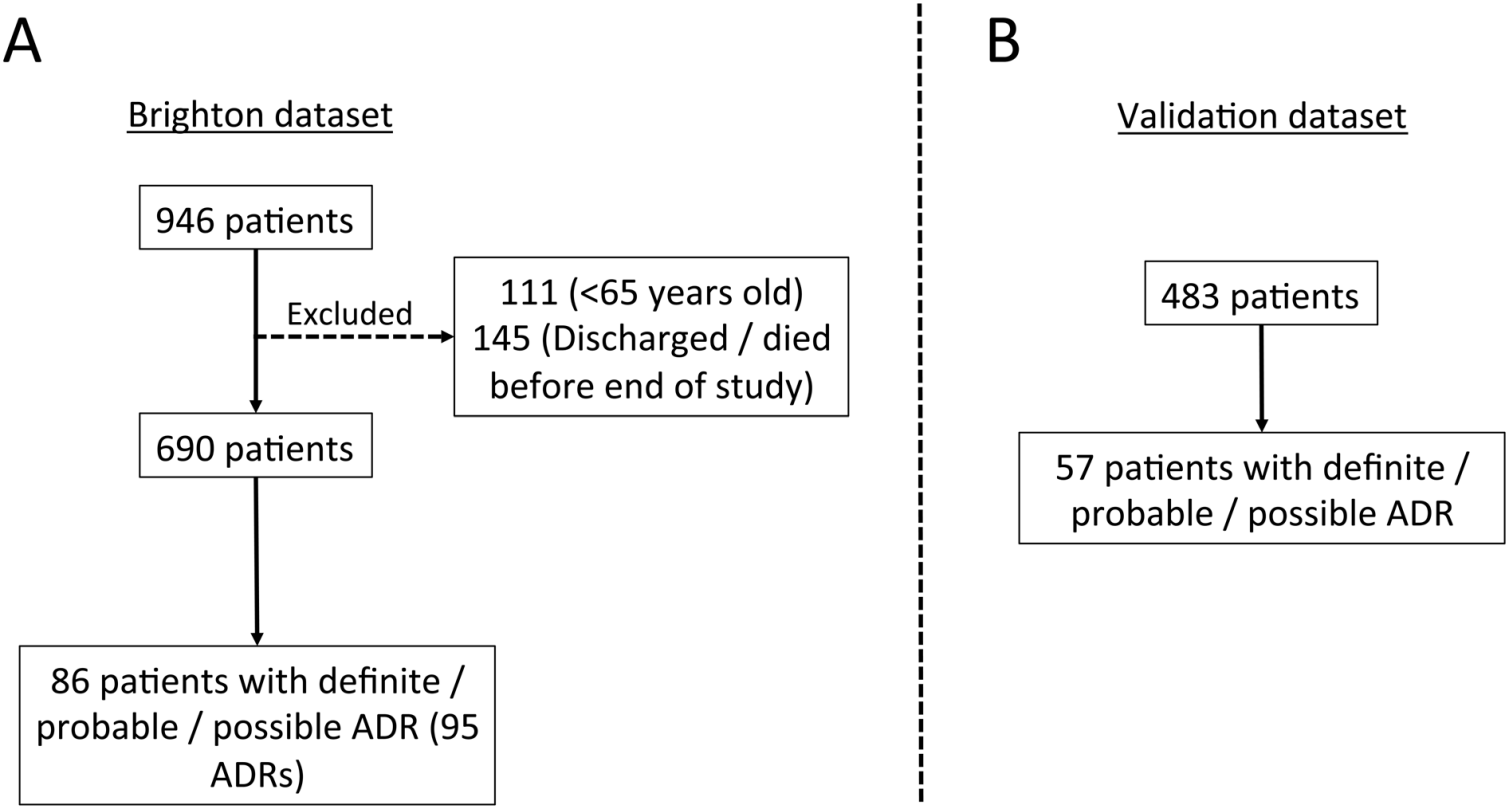
Recruitment diagram for the Brighton and Validation datasets.

The initial *Brighton dataset* study population comprised 946 patients. Two-hundred and fifty-six of these patients were excluded in the final analysis because they were either younger than 65 years old (111 {43%} patients), or were rapidly discharged or died before the end of the study period (145 {57%} patients). Patient recruitment was further hindered by an outbreak of *Norovirus* and *Clostridium difficile* infection on two of the study wards which restricted access to patients on the grounds of infection control. Consequently, 690 patients formed the final *Brighton dataset* (**Figure** **1**).

The mean age of patients in the *Brighton dataset* was 84.3 years (range 65–103; median 85; inter-quartile range 81–89), with a larger proportion of the cohort being female (61%) than male. The mean number of regular medications (taken daily, including topical preparations) taken on admission by these patients was 5.9 (range 0–18; median 6; IQR: 3–8). The presence and burden of co-morbidities was high, with the mean number of recorded co-morbidities in this study sample being 8.03 (range 1–17; median 8; IQR: 6–10). **Table 2** provides details of the common co-morbidities found in this patient group. Forty one percent of patients were widowed, 35% married, 61% lived in a house or flat and 10.6% were living in nursing homes. Almost 57% (n = 381) of the total patients were living alone and half of them required a degree of social support.

**Table 2:** Patient characteristics and co-morbidities in the Brighton and validation (European) data sets.

	No (%) of patients^ψ^	
	Median (interquartile range)	
Demographics	*BADRI dataset* (n = 690)	*Validation dataset* (n = 483)
Age (yr)^ψ^	85 (81–89)	80 (75–86)
Gender (Female)	419 (61)	279 (57.8)
Ethnic Origin (White-British)	607 (88)	
**Clinical**		
Length of Stay^ψ^	12 (7–19)	10 (6–17)
Co-morbidities^ψ^	8 (6–10)	
Barthel Activity of daily Living^ψ^	19 (14–20)	
Katz Activity of Daily Living^ψ^		1 (0–4)
Glasgow Coma Scale^ψ^	15 (14–15)	
Cognition (AMTS)^ψ^	6 (3–9)	
Cognition (MMSE)^ψ^		26 (22–28)
Previous Hospital Admission	168 (25)	
**Social**		
Smoking	59 (8)	
Alcohol	183 (41)	
Living Alone	383 (57)	
**Drug Related**		
Number of regularmedications on admission^ψ^	5 (3–7)	5 (4–8)
Number of regularmedications on the ward^ψ^	7 (5–10)	9 (6–14)
Previous drug allergies	149 (22)	
Previous History of ADR	263 (38)	173 (35.8)
**Co-morbidities**		
Hypertension	502 (72.8)	305 (63.1)
Infection (UTI/Chest infection)	303 (43.9)	125 (25.9)
Anaemia	283 (41)	135 (28)
Arthritis/Osteoarthritis	280 (40.6)	
Renal impairment(<60 mLs/min)	248 (35.9)	66 (13.7)
Fall	209 (30.3)	114 (23.8)
Depression	176 (25.5)	
Confusion	176 (25.5)	
Ischemic Heart Disease	159 (23)	
Atrial Fibrillation	156 (22.6)	
Asthma/COAD	135 (19.6)	
Malignancy	133 (19.3)	
Diabetics	115 (16.7)	131 (27.1)
Previous stroke	115 (16.7)	
Previous TIA	110 (15.9)	
Osteoporosis	86 (12.5)	
Hyperlipidaemia	284 (12.2)	135 (28)
Congestive Heart Failure	70 (10.1)	67 (13.9)
Dementia(other than Alzheimer)	74 (10.7)	
Alzheimer	25 (3.6)	
Liver diseases	7 (1)	30 (6.2)

Abbreviations: Mini-Mental State Examination, MMSE; Abbreviated Mental Test Score, AMTS; Unrinary Tract Infection, UTI; Chronic Obstructive Airways Disease, COAD; Transient Ischaemic Attack, TIA; Adverse Drug Reaction, ADR.

Following the initial assessment of patient histories, 95 cases of ADR, in 86 patients were identified in the *Brighton dataset* (12.5%). An assessment of causality was then conducted using the Hallas algorithm, which rated 23 of these cases as definite (24.2%), 47 as probable (49.5%), and 25 cases as possible (26.3%). All 95 events also received confidence rating of >50% (score >3) and so were retained for subsequent analysis of the *Brighton dataset*. The drugs classes with highest frequency of reported ADRs were cardiovascular, analgesic and anti-diabetic. A detailed analysis of the types, severity, and preventability of the ADRs have been published elsewhere [10].

### Development of the BADRI risk model

Thirteen variables showing a significant difference in prevalence between ADR and non-ADR patients were identified from the univariate analysis and taken forward to the next stage of model development (**Table 1**). A further 5 variables showing minimal association in this dataset (p<0.25), but which had been identified as important in predicting ADR risk by other studies, were also included at this stage. This differs from the methodology used by GerontoNet, but supplementing the model with additional clinically plausible variables is nonetheless an accepted technique [20]. For the continuous predictor variables, multicolinearity tests found no significant associations, and so these variables were retained in the model. Of the remaining binary variables, ten were found to be correlated and were subsequently discarded. This resulted in a total of 8 variables being taken forward to the multivariate analysis stage (**Table 1**).

Application of both the backward and forward stepwise elimination procedure during multivariate analysis identified 5/8 variables that contributed significantly to the risk of developing an ADR (Wald test scores, p<0.1; **Table 3**). The Hosmer-Lemeshow test showed that the fit was satisfactory (χ^2^ = 4.196, 7 degrees of freedom, p = 0.757); however, the Nagelkerke R^2^ value was low (0.16), indicating a small effect size. Although small, the model provides more information about ADR risk compared to a model with no predictors. All 5 variables were subsequently used to form the final *BADRI* risk model.

**Table 3:** Results of multivariate analysis.

Final variables	B	S.E	Wald	Sig.	OR	95% CI
**Hyperlipidemia**	1.199	0.309	15.093	<0.001	3.316	1.811–6.072
**Number of medication ≥8**	1.194	0.274	18.922	<0.001	3.300	1.927–5.651
**Length of stay ≥12 days**	0.819	0.267	9.441	0.002	2.269	1.345–3.826
**Use of anti-diabetic agents**	0.645	0.309	4.352	0.037	1.906	1.040–3.493
**High WCC on admission**	0.437	0.254	2.953	0.086	1.548	0.940–2.548
**Constant**	-3.628	0.316	131.769	1	0.000	0.027

(WCC = White Cell Count).

### The ability of the BADRI risk model to predict ADR in the Brighton dataset

**Figure 2:**
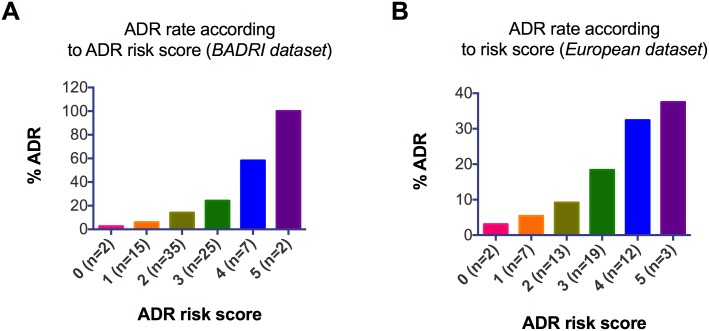
ADR rate according to ADR risk score. The BADRI risk model was applied to all 690 patients from the Brighton dataset (A), and 483 patients from the European dataset (B). The ADR rate is calculated as the proportion of patients in each scoring category that suffered an ADR. For both datasets there is a general increase in the ADR rate as the risk score increases.

Following development of the *BADRI* risk model, we attributed an ADR risk score to each patient in the *Brighton dataset* based of the number of variables present. For simplicity, an equal weighting of 1 was attributed to each variable. The range of the risk scores for patients in the *Brighton dataset* was 0–5, with a mean of 1.5, standard deviation of 1.05, and a median of 1. When grouped according to BADRI risk model, there was a clear relationship between risk score, and the proportion of ADRs (**Figure 2A**).

To maximise the accuracy of the model in predicting ADRs in patients, we performed a further analysis to determine the most appropriate cut-off risk score. This was done by calculating the sensitivity (true positive) and specificity (true negative) of the model using each risk score (0–5) as a cut-off value, and then calculating Youden’s Index (**Table 4**); the greater the index the more discriminatory the model at the specified cut-off value. Youden’s Index was found to be greatest when the cut-off score was >1 (J = 0.36; sensitivity and specificity were 80%, and 55% respectively. Performance of the ADR risk model was also assessed from a calculation of the area under the receiver operator curve (AUROC). The model was found to have an AUROC of 0.74 (95% CI 0.68 to 0.79, **Figure S1**), suggesting that the ability of the BADRI model to predict ADRs is better than chance alone (0.5).

**Table 4:** Accuracy of the BADRI risk model as applied to the *Brighton dataset* using various cut-off values (risk scores).

RiskScore	Patientswith ADR	Patientswithout ADR	Sensitivity	Specificity	Youden’sindex (J)	1-specificity
>0	84	486	0.98	0.20	0.17	0.80
>1	69	269	0.80	0.55	0.36	0.45
>2	34	89	0.40	0.85	0.25	0.15
>3	9	14	0.10	0.98	0.08	0.02
>4	2	0	0.02	1.00	0.02	0.00

Note Youden’s Index is largest when the cut-off value is >1 (with sensitivity of 80% and specificity of 55%).

### Validation

Validation of the *BADRI* risk model was conducted on patient data gathered from 4 European centres (the *European dataset*). Four-hundred and eighty-three patients were recruited, with a mean age of 80.3 years (range 65–99; median 80; inter-quartile range 75–86), and mean number of drugs taken during hospital stay of 11±7.0. ADRs were observed in 56 patients (11.6%).

All variables that form the *BADRI* risk model, with the exception of raised white cell count were associated with increased rates of ADR in patients that comprise the *European dataset*. When grouped according to ADR risk score, a similar relationship between risk score and the proportion of ADRs, to that seen in the *Brighton dataset* was observed (**Figure 2B**). As with the *Brighton dataset*, a cut-off score of >1 provided the best discrimination between individuals that progressed to suffer an ADR and those that did not (J = 0.26, sensitivity and specificity values of 84% and 43% respectively, **Table 5**). The BADRI risk model also demonstrated an acceptable capacity to discriminate in the *European dataset*, with an AUROC of 0.73 (95% CI 0.66–0.80, **Figure S1**), indicating that it is statistically useful in predicting ADR not only when applied in the local area (Brighton), but also in other populations.

**Table 5:** Accuracy of the BADRI risk model as applied to the *European dataset* using various cut-off values (risk scores).

RiskScore	Patientswith ADR	Patientswithout ADR	Sensitivity	Specificity	Youden’sindex (J)	1-specificity
>0	53	342	0.95	0.20	0.15	0.80
>1	45	232	0.80	0.46	0.26	0.54
>2	26	108	0.46	0.75	0.21	0.25
>3	10	22	0.18	0.95	0.13	0.05
>4	1	3	0.02	0.99	0.01	0.01

Note Youden’s Index is largest when the cut-off value is >1 (with sensitivity of 80% and specificity of 46%).

## Discussion

We have produced an ADR risk prediction model, based on 5 clinical variables, which is able to identify older patients that have an increased likelihood of developing an ADR. The model was developed using data gathered from a population of patients in the UK with a median age of 85 years, which makes this the first study to address ADR risk specifically in the oldest old, a proportion of the US and UK population which is increasingly growing. The *BADRI* risk prediction model has been successfully validated in older patients (although not the oldest old) from hospitals across continental Europe, showing that it is a reasonably robust model, that may be applied to patients from other geographical locations, and perhaps from different healthcare systems with a similar demographic. The final BADRI risk prediction model contains only a small number of variables, and each one is given the same score (1 in each case), making it relatively simple to use. This may make its adoption into routine clinical practice easier.

The BADRI score was found to have acceptable goodness of fit and good discrimination performance when applied to both *Brighton* and *European* datasets. An AUROC of greater than 0.7 for the model showed that it has acceptable discrimination capacities for patient with ADRs as compared to patients who did not experience an ADR. The sensitivity of 80% indicates that the model, in its present form, is a satisfactory predictor of ADR. However, the low specificity (46%) means that the model may incorrectly label patients ‘at risk of an ADR’ who will not ordinarily go on to experience such an event. A false positive in this instance may have implications for the patient’s management. For example, important treatments may unnecessarily be withheld based on inaccurate risk stratification. In addition, inappropriately labelling patients as risky when they are not may result in the inefficient use of resources due to increased monitoring.

A direct comparison of the BADRI score with the recently developed GerontoNet risk score [8] show that both perform similarly on levels of discrimination (0.71, GerontoNet vs. 0.74, BADRI). The sensitivity value for the BADRI model during development was 80%, exceeding that of GerontoNet at 68%, indicating that BADRI performs better at detecting patients that go on to have an ADR. However, its specificity value (or true negative) of 55%, was lower than GerontoNet's at 65%. It must be noted however, that GerontoNet only considered cases of definite and probable ADRs for inclusion in their study, whereas possible ADRs were included in the present study. Other studies which have attempted to develop predictive models for adverse drug events have not been externally validated which restricts their applicability to other populations.

Much work has been conducted over recent years to identify clinical risks associated with ADR. Interestingly, a number of these risks were not found to be significant predictors of ADR in this current study. For example, well known factors such as age [23], previous history of ADR, gender [24], heart failure [8], prior bleeding on admission [25], renal impairment [26], the use of certain drug classes [27], and abnormalities in certain laboratory parameters [6] were not retained in our final model. The omission of these previously identified risk factors could possibly be explained by the inclusion criteria, which recruited a broader range of ages. Of the variables included in our final model, the number of medications prescribed during inpatient stay was the only variable that has been consistently identified as an ADR risk in all other validated studies. One variable previously identified by O’Connor et al to predict ADR risk is that of potentially inappropriate medicines. These were not included in this study as this variable was identified after our study had been conducted [9].

This is the first study to include total length of stay, hyperlipidaemia and white cell count as ADR risk factors. Length of stay may be a proxy measure for the severity of the patients' underlying illness, perhaps reflecting an increase in the number of prescribed medicines (with associated risk of drug interactions). However, the fact that the number of medications prescribed is also included as a risk factor in the BADRI model, and that, when tested, no association between the two variables was found, suggests that they represent different mechanisms. Alternatively, an increased length of stay may reflect a deterioration in the patients clinical state, and a change in the pharmacokinetic (for example, renal and hepatic function, nutritional status) or pharmacodynamic (tissue sensitivity) profile of the medication, leading to altered drug levels or response. It is also conceivable that a prolonged hospitalisation could increase the likelihood of experiencing, and detecting, an ADR, i.e. *a posteriori* observation, which questions the value of this variable as a useful predictor.

We also found that a diagnosis of hyperlipidaemia significantly increased the likelihood of developing an ADR. The reason for its identification as a risk factor has yet to be determined, but may simply reflect the fact that individuals with a history of an abnormal lipid profile are at higher risk of cardiovascular disease, and as a consequence, may be receiving multiple drug therapies. An association between a high white blood cell count on admission and ADR risk was another novel risk factor identified in the current study. This may reflect that the patient is suffering from an infection or inflammatory condition (for example, post myocardial infarction or pulmonary embolism) which requires the subsequent use of potential harmful antibiotics or cardiac medications. Regardless of the cause of raised WCC, it is nonetheless a marker for increased susceptibility to ADR, and will alert the clinician to the patients increase risk of developing an adverse reaction to a medicine.

Although there are several advantages of the BADRI score in predicting ADR risk, there are of course some limitations that need to be addressed. Causality assessment for ADR in the testing dataset was conducted using Naranjo’s algorithm, however, during the initial development of the model (*Brighton dataset*), the Hallas criteria was utilised to determine causality. It is therefore plausible that the use of different criteria might affect the outcome of the study. However, in addition to the Hallas criteria, we asked reviewers to rate causality as being a drug cause on a Likert scale, and also included all classifications of ADR (definite, probable and possible) in our final model, so we can be reasonably confident that ADR were classified appropriately. Another limitation of this study is the generalizability of the findings. The current tool was validated in four European countries and may not therefore be applicable to countries outside Europe.

The results of this study may also be subject to a small extent of information bias, especially in the validation study group. Information bias occurs when data collected from patients is pursued more aggressively in one group or recorded differently than in another. In addition, there might also be a tendency to recall bias (another form of information bias). Older patient populations have a higher incidence of memory impairment which could affect the quality of the data collected. However, careful evaluation and extraction of information from the patient notes reduces the chance of recall bias in this study.

Although this study has identified a number of variables which predict ADR in some older patients, as a model, the BADRI risk score needs further development before it can be adopted for use in routine clinical practice. The reasons for this are (1) we have yet to establish whether clinical judgement alone is more accurate than the BADRI score at identifying those individuals who go on to develop an ADR (2) despite BADRI being relatively simple to use by our experienced researchers, we have yet to test the usability of the model by clinicians (3) we have yet to determine whether introducing this model into routine practice would improve the safety profile of medication use in older patients, and also whether there are any humanistic and cost implications.

## Conclusion

The development of the BADRI score, which consists of five variables for predicting ADR in older patients, was chosen using a stepwise selection procedure. The predictors were chosen based on the combination of statistically significant tests and also by consideration of the biological plausibility and conceptual appropriateness of the variables. External validation conducted in different locations retained the fitting and also the predictive power of the model which ensure the generalisability of the score. The BADRI score consists of different risk predictors compared to the GerontoNet risk score besides the number of medication, yet it offers a scoring system that is simple to apply with limited computational involvement, whilst providing the same discrimination performance.

## Supporting Information

Figure S1 Receiver Operator Curves for the Brighton dataset (A) and the Validation dataset (B). The AUC are 0.737±0.028 and 0.727±0.034, and the asymptotic significance 0.000 (0.683–0.791 95% CI) and 0.000 (0.660–0.794 95% CI) for the Brighton and Validation datasets respectively.
(TIF)
