# Supplementary figures and images for: Development and Validation of a Risk Model for Predicting Adverse Drug Reactions in Older People during Hospital Stay: Brighton Adverse Drug Reactions Risk (BADRI) Model
Source: PLoS One. 2014 Oct 30;9(10):e111254. doi: 10.1371/journal.pone.0111254 (PMC4214735; doi:10.1371/journal.pone.0111254)

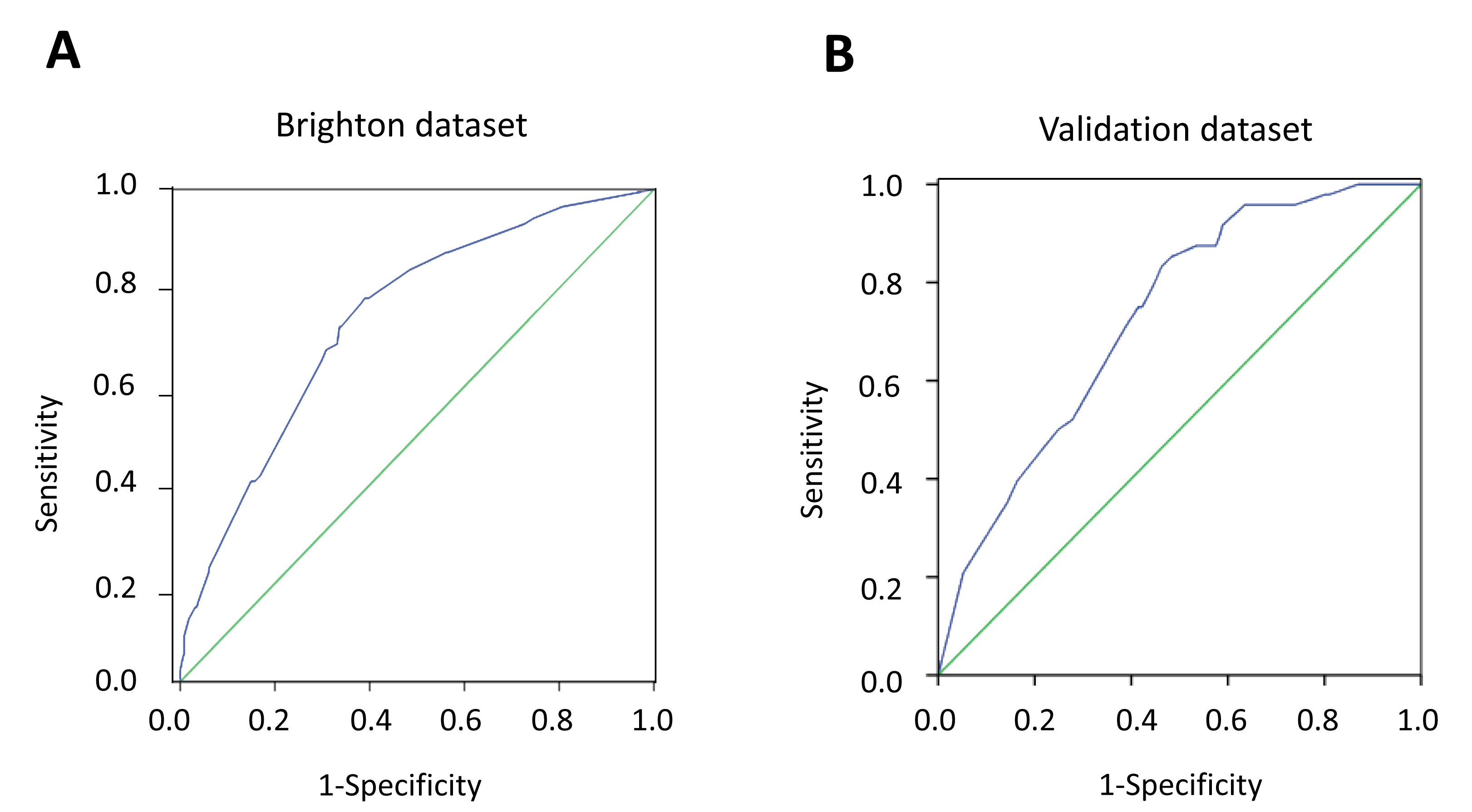

Supplement: Figure S1 — Receiver Operator Curves for the Brighton dataset (A) and the Validation dataset (B). The AUC are 0.737±0.028 and 0.727±0.034, and the asymptotic significance 0.000 (0.683–0.791 95% CI) and 0.000 (0.660–0.794 95% CI) for the Brighton and Validation datasets respectively. (TIF) [file pone.0111254.s001.tif]
